# 980-nm diode laser excision of a giant pregnancy epulis

**DOI:** 10.12669/pjms.38.3.5102

**Published:** 2022

**Authors:** Lifeng Li, Yanming Liu

**Affiliations:** 1 Lifeng Li, Department of Stomatology, The First People’s Hospital of Fuyang Hangzhou, Hangzhou 311400, China; 2 Yanming Liu, Department of Oral and Maxillofacial Surgery, The Second Affiliated Hospital of Zhejiang University School of Medicine, Hangzhou 310009, Zhejiang, China. Department of Stomatology, The First People’s Hospital of Fuyang Hangzhou, Hangzhou 311400, China

**Keywords:** Diode laser, Epulis, Pregnancy

## Abstract

The pure pregnancy epulis is mostly vascular, and generally shrinks or disappears with the drop in estrogen levels following delivery. However, the fibrous epulis or granulomatous epulis may enlarge rapidly in the early stages of pregnancy, necessitating surgical resection after pregnancy. This report described a 25-year-old patient with a post-term pregnancy. She had a lump on the buccal side of left mandibular molar region that was associated with intermittent hemorrhage and eating difficulty. Since the patient feared the conventional surgery, the 980-nm diode laser excision was performed to remove the epulis two weeks after delivery, with the involved teeth preserved. The new technique of 980-nm diode semiconductor laser may be a potential good option to treat pregnancy epulis, with less discomfort and involved teeth preserved.

## INTRODUCTION

Epulis is derived from the connective tissues of periodontal ligament and jaw alveolar process, and is considered to be a reactive proliferation caused by mechanical stimulation and chronic inflammatory stimuli.^1^ Since the epulis has no tumor-specific structure, it is not a true neoplasm. However, the epulis has the appearance and biological behavior of a tumor, including relapse after resection and responsiveness to endocrine stimulation. The pregnancy epulis is prone to relapse, but shrinks or ceases to grow after delivery.^2^ The epulis may be managed with surgical measures including electrocoagulation, and microwave or laser treatment, as well as immediate teeth replantation after removal of affected teeth and resection of the epulis, or with non-surgical measures like intratumoral injection of a mixed solution of pingyangmycin and dexamethasone.

At present, the surgery is still the primary choice, and it includes resection of epulis and, to reduce relapse, removal of the affected teeth as well.^3^ The removal of teeth may destroy the integrity of the dentition, reduce the masticatory function, affect the speech, and reduce the quality of life. Pregnant women are often afraid of surgery and tooth extraction, and prefer to a mild alternative. Semiconductor laser is a new and effective method to manage oral soft tissue lesions. In this study, we reported a case of giant pregnancy epulis treated with 980-nm diode semiconductor laser surgery two weeks after delivery.

## CASE PRESENTATION

**Fig.1:**
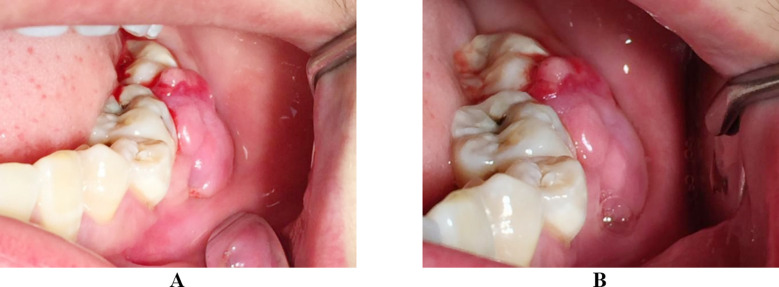
(A) A pregnancy epulis in the left mandible in the patient at 37 weeks of gestation. (B) The pregnancy epulis remaining in the left mandible in the patient two weeks after delivery.

A 25-year-old patient with a post-term pregnancy visited our hospital due to hemorrhage of epulis in the left lower posterior tooth region. The epulis was only at the size of a soybean before pregnancy, and it gradually increased during pregnancy, especially in the last month. Since the epulisrepeatedly bled and affected eating in the past weeks, it was necessary to be treated. A tumor-like mass was found on the buccal side of the left mandibular molar region between the lower left second premolar and the second molar on physical examination; the mass was irregular in appearance and measured 1×2.5×0.8 cm, and tended to bleedon its distal surface. Her oral hygiene was poor. The lower left mandibular second molar showed the first degree loosening, while the first molar second degree loosening. Since the date of delivery was close, no other special measure than mouth rinse with chlorhexidine was administered for anti-infection. The patient returned two weeks after a spontaneous delivery, complaining of increased tumor bleeding and discomfort due to swelling (Fig.1). Oral panoramic X-rays showed alveolar bone reabsorption between the lower left first molar and second molar.

**Fig.2:**
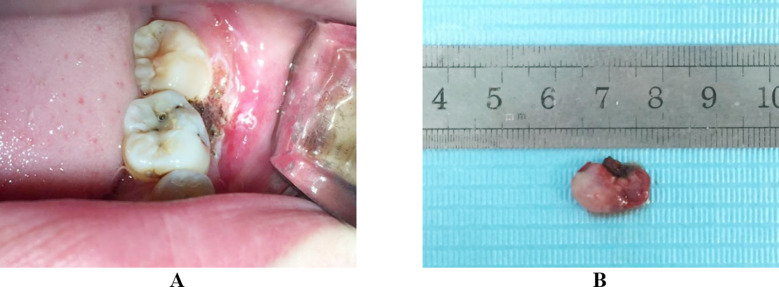
(A) Intraoperative view of the wound after removal of the pregnancy epulis by 980-nm diode laser. (B) The pregnancy epulis resected by 980-nm diode laser.

**Fig.3:**
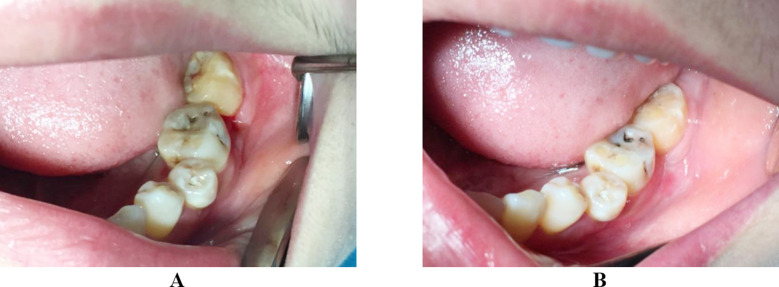
(A) The wound had been completely healed at the postoperatively two week. (B) No evidence of relapse in the one-year follow up and the involved teeth remained.

After fully informed of the benefits and complications of the optional measures to manage the epulis, the patient preferred to the 980-nm semiconductor laser surgery, with the preservation of the involved teeth. We used SIRO Laser Advance semiconductor laser equipment that was produced in Bentheim, Germany. Equipment parameters were λ = 980±15 nm and P_max_ = 14 W. Parameters we selected at the treatment were 5-15 J/cm^2^, 20-60 ms of the pulse width, and 1 mm of the spot diameter. We completed the resection at one time, with laser resection time of five minutes and the cumulative energy of 120 J. Under local infiltration anesthesia, the excision was made using 300 μm fiber, with power of 6 W and ratio of 50%, frequency of 10 Hz, average power of 3 W, and pulse mode of 4 W. No postoperative medication was prescribed (Fig.2). The surgical site had been completely healed at the two weeks. No evidence of recurrence was observed in the one-year follow up. More surprisingly, the teeth mobility was significantly decreased (Fig.3).

## DISCUSSION

The occurrence and development of epulis is closely related to endocrine activity. Women at pregnancy are especially prone to recurrence of epulis.^4^ Epulis can undermine alveolar bone with tumor growth, causing tooth looseness and displacement. The primary method to treat epulis is conventional surgery, which includes resection of epulis, extraction of affected teeth, and curettage of periodontal ligament, periosteum and adjacent bone tissues as well. In this way, the recurrence rate may be effectively controlled. However, the sacrifice of the involved teeth and the surrounding alveolar bone tissues may negatively affect the post-operative quality of life. A non-surgical approach is intratumoral injection of a mixed solution of pingyangmycin and dexamethasone, which does achieve some therapeutic effect.^5^ However, it is not suitable for nursing women.

Diode lasers have the widest range of application in dentistry. They are used in endodontics and periodontics for their ability to reduce bacterial load, and also in surgery, as well as soft tissue and peri-implantitis treatment.^6^ For diode lasers surgery, it is optional to apply wavelengths of 810 or 980 nm. Study has shown that the bactericidal effect of an 810-nm diode laser is better than that of a 980-nm laser, because of greater penetration into tissue.^7^ As shown in this case, the 980-nm diode laser surgery might have some advantages over the conventional surgery of pregnancy epulis resection. Firstly, the 980-nm diode laser surgery causes little trauma and almost no bleeding. Secondly, this technique makes it possible to preserve the teeth within the surgical field. The over-one-year-long follow-up had showed no evidence of recurrence due to the preservation of the involved teeth. Thirdly, since it is necessary to removal the surrounding periodontal tissues, alveolar bone and teeth, the whole procedure becomes much simple.

## CONCLUSION

Compared with the traditional surgical methods, the semiconductor laser surgery does not require suturing of wound and causes less postoperative discomfort, which has unparalleled advantages in oral soft tissue surgery. The new technique of 980-nm diode semiconductor laser may be a potential good option to treat pregnancy epulis, causing less discomfort and preserving the involved teeth. However, more clinical cases are required to confirm its reliability.
